# Life Cycle Assessment of Coastal Enhanced Weathering for Carbon Dioxide Removal from Air

**DOI:** 10.1021/acs.est.2c08633

**Published:** 2023-04-03

**Authors:** Spyros Foteinis, James S Campbell, Phil Renforth

**Affiliations:** Research Centre for Carbon Solutions, School of Engineering and Physical Sciences, Heriot-Watt University, Edinburgh EH14 4AS, UK

**Keywords:** ocean alkalization, enhanced silicate weathering in coastal systems, enhanced rock weathering, negative emissions technology, ocean alkalinity enhancement

## Abstract

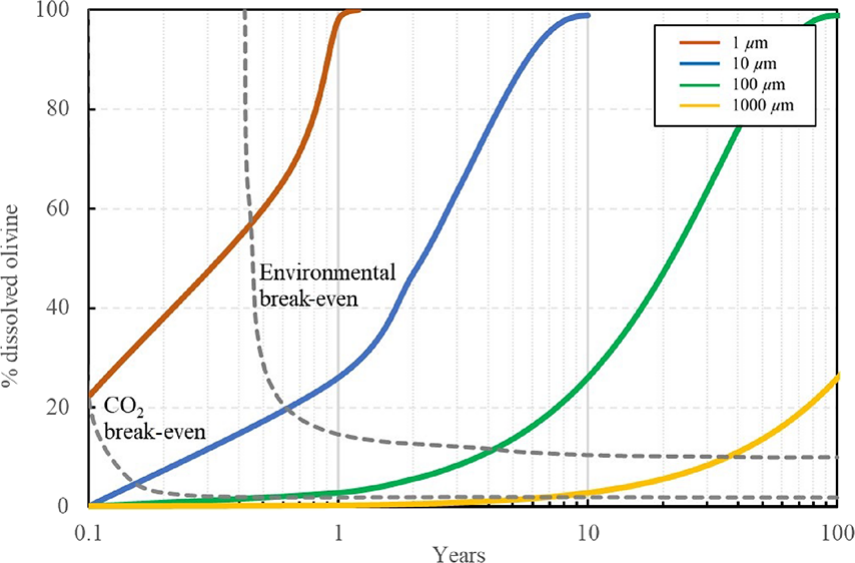

Coastal enhanced weathering (CEW) is a carbon dioxide removal (CDR) approach whereby crushed silicate minerals are spread in coastal zones to be naturally weathered by waves and tidal currents, releasing alkalinity and removing atmospheric carbon dioxide (CO_2_). Olivine has been proposed as a candidate mineral due to its abundance and high CO_2_ uptake potential. A life cycle assessment (LCA) of silt-sized (10 μm) olivine revealed that CEW’s life-cycle carbon emissions and total environmental footprint, i.e., carbon and environmental penalty, amount to around 51 kg CO_2_eq and 3.2 Ecopoint (Pt) units per tonne of captured atmospheric CO_2_, respectively, and these will be recaptured within a few months. Smaller particle sizes dissolve and uptake atmospheric CO_2_ even faster; however, their high carbon and environmental footprints (e.g., 223 kg CO_2_eq and 10.6 Pt tCO_2_^–1^, respectively, for 1 μm olivine), engineering challenges in comminution and transportation, and possible environmental stresses (e.g., airborne and/or silt pollution) might restrict their applicability. Alternatively, larger particle sizes exhibit lower footprints (e.g., 14.2 kg CO_2_eq tCO_2_^–1^ and 1.6 Pt tCO_2_^–1^, respectively, for 1000 μm olivine) and could be incorporated in coastal zone management schemes, thus possibly crediting CEW with avoided emissions. However, they dissolve much slower, requiring 5 and 37 years before the 1000 μm olivine becomes carbon and environmental net negative, respectively. The differences between the carbon and environmental penalties highlight the need for using multi-issue life cycle impact assessment methods rather than focusing on carbon balances alone. When CEW’s full environmental profile was considered, it was identified that fossil fuel-dependent electricity for olivine comminution is the main environmental hotspot, followed by nickel releases, which may have a large impact on marine ecotoxicity. Results were also sensitive to transportation means and distance. Renewable energy and low-nickel olivine can minimize CEW’s carbon and environmental profile.

## Abstract 2

LCA advised on CEW’s environmental aspects, providing avenues to sustainably scale up the process and maximize atmospheric CO_2_ drawdown and environmental credits.

## 1 Introduction

Over thousands to millions of years, the earth’s climate is influenced by the natural weathering of land surface minerals,^1^ whereby the alkalinity that is released by the weathering of calcium (Ca)- and magnesium (Mg)-bearing rocks reaches the oceans and uptakes atmospheric carbon dioxide (CO_2_).^2^ This is a result of the equilibrium between the concentration of CO_2_ in the atmosphere and the seawater’s total dissolved inorganic carbon (DIC) content.^3^ As a result, ∼1 Gt of atmospheric CO_2_ is naturally taken up each year and stored in surface ocean waters, mainly as an inert bicarbonate (HCO_3_^–^) and to a lesser extent as carbonate (CO_3_^2–^) ions.^4^ There, carbon remains safely stored for up to hundreds of thousands of years^5^ and eventually precipitates, typically forming carbonate minerals.^6^ Currently, the oceans hold ∼38,000 Gt of carbon as DIC, which is 40 times higher than the mass of carbon in the atmosphere.^7^ However, the rates of the weathering reactions that govern the uptake of atmospheric CO_2_, such as the dissolution of olivine ((Mg, Fe)_2_SiO_4_) in water (H_2_O) to Mg, iron (Fe), orthosilicic acid (H_4_SiO_4_), and bicarbonate (eq 1) are relatively slow^8^ and therefore cannot passively mitigate the increasing anthropogenic (surplus) CO_2_ emissions. The increased anthropogenic CO_2_ emissions have also affected the oceans’ buffer capacity and carbonate mineral saturation states, causing unprecedented changes in the ocean chemistry and also leading to ocean acidification.^6,9^
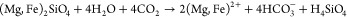
1

As such, research has now focused on enhancing the rates of these reactions (eq 1), mainly through the mining, crushing, and spreading of silicate rocks either (i) in land (typically in humid tropics where weathering reactions are faster),^2^ a process known as terrestrial enhanced weathering, or simply enhanced weathering (EW),^10^ or (ii) in the coastal zone, where the natural wave and tidal forces will catalyze the weathering, a process known as coastal enhanced weathering (CEW).^3,11^ Both have emerged as promising carbon dioxide removal (CDR) approaches,^11,12^ with additional benefits of nutrient addition and mitigating acidification in soils and/or the ocean.^6^ Specifically, part of the produced alkalinity in EW will be transported by streams, rivers, and subsurface waters to the oceans.^10^ However, large amounts of alkalinity released at scale^13^ could have adverse effects, especially since freshwater ecosystems are sensitive to changes in the pH levels^14^ and might be already affected by salinity and alkalinity due to anthropogenic activities and accelerated weathering.^15^ This limitation is less constraining in CEW; since the oceans are affected by acidification,^16^ seawater’s average pH is much higher, i.e., 8.1,^17^ and marine carbonate chemistry is less sensitive to alkalinity addition at a global scale.^18^ Therefore, in CEW, the added alkalinity can rapidly dilute,^19^ and this could also help prevent the formation of secondary minerals, such as clays, via reverse weathering.^20^ For these reasons, research has also focused on CEW, particularly when using olivine, which combines widespread abundance with fast dissolution.^21^ However, unlike EW^22^ and other ocean-based CDR approaches such as ocean liming,^23^ CEW’s environmental sustainability remains largely unknown.

Here, the life cycle assessment (LCA) methodology was employed to explore CEW’s environmental impacts and climate change mitigation benefits when using olivine. Furthermore, sensitivity analyses were carried out, focusing on olivine particle size and composition, as well as on the effect of electricity mix for comminution and transportation means and distance. Overall, this LCA study is one of the first attempts to comprehensively identify the life-cycle carbon emissions and environmental impacts and benefits of this ocean-based CDR approach. Results can inform the sustainable co-deployment of CEW with other CDR approaches, such as EW,^22^ ocean liming,^23^ direct air capture and storage (DACS),^24^ ocean fertilization, bioenergy with carbon capture and storage (BECCS), and afforestation/reforestation,^25^ and thus maximize CO_2_ drawdown.

## 2 Materials and Methods

CEW’s environmental sustainability was examined using the LCA methodology^26,27^ and employing the software program SimaPro (version 9.4.0.2). A base scenario was considered, whereby silt-sized (10 μm) olivine, with a 0.263% nickel (Ni) concentration,^28^ is spread in Europe’s waters (average water temperature 15 °C). Different scenarios for the effect of olivine particle size, composition and transportation and the effect of electricity mix were also examined.

### 2.1 Electricity Mix, Olivine Composition, and Transportation Means and Distance

Electricity generation is not currently fully decarbonized and is dependent on fossil fuels. Comminution to small particle sizes can be energy-intensive,^29^ increasing CEW’s carbon and environmental footprint. Replacing fossil fuel-dependent electricity with renewable electricity can improve the environmental sustainability of energy-consuming systems,^30^ and therefore, this was considered in the sensitivity analysis.

Furthermore, olivine can contain elements/impurities,^31,32^ which, depending on their nature and concentration, may be benign, beneficial, or harmful to local ecosystems. For example, dissolved silicon (DSi) and Fe are key nutrients for phytoplankton that are lacking in many parts of the oceans.^21^ Therefore, their possible indirect addition through CEW^10^ could stimulate the biological carbon pump, but more research is required.^21^ Alternatively, Ni and chromium (Cr) can be present in olivine-rich rocks,^33^ which, if found in elevated concentrations, can cause harm to coastal ecosystems and thus hamper CEW’s eligibility as a CDR.^34^ Ni is typically well distributed within olivine’s mineral matrix;^28,35^ however, this is not the case for Cr, which is concentrated in insoluble Fe-rich particles,^33^ implying low bioavailability and lower ecotoxicological concerns compared to Ni.^34^ For this reason, focus was placed here on the effect of Ni concentration as a representative impurity of olivine that can be released in coastal environments. Finally, it has been identified that overland transportation can be an important factor influencing EW’s environmental sustainability,^22^ and this was also examined herein.

### 2.2 Particle Size and Dissolution Rates in Coastal Environments

Equation 1 suggests that 1 mole of dissolved olivine can sequester 4 moles of CO_2_, i.e., 1.25 kg CO_2_ per kg of olivine.^28^ However, the overall CO_2_ sequestration potential is 20% lower when accounting for buffering within the carbonate system^13^ and may be lower still when considering inefficiencies in air–sea gas exchange.^36^ Furthermore, olivine-rich silicate rocks like dunite,^10^ an ultramafic intrusive igneous rock that belongs to the peridotite group and contains around 90% of olivine,^37^ will likely be used in CEW rather than pure olivine. Therefore, a conservative assumption was made and peridotite’s CO_2_ sequestration potential was used (0.8 kg of CO_2_ per kg of rock).^38^ No adjustments were made for inefficiencies in air–gas exchange given that these are location-specific and may not be applicable to weathering in dynamic coastal environments. Furthermore, apart from seawater pH and temperature,^39^ the olivine dissolution rate and, by extension, the corresponding atmospheric CO_2_ uptake rates greatly depend on the size (specific surface area) of the particles that will be weathered since larger particles will dissolve significantly more slowly than smaller ones.^40^

Specifically, the dissolution rate greatly depends on the mean particle size since, for example, at 25 °C seawater temperature, the 10 and 300 μm olivine can take, on average, 23 and 700 years, respectively, to dissolve.^41^ Finely ground (≤1 μm) olivine can remain suspended in seawater and dissolve in short timescales;^21^ however, most likely, such a high degree of grinding would be associated with significant engineering challenges (comminution, transportation, and spreading), while it might also induce particulate air pollution and silt pollution (turbidity) in coastal waters. Here, olivine dissolution rates were estimated using the shrinking core model,^41^ which suggests that at 15 °C water temperature the 10 μm olivine (base scenario) will dissolve within 10 years. Particle sizes as low as 1 μm to as high as 1000 μm were considered in the “Sensitivity Analysis” section. In the former, coastal dissolution is rapid (few months/years),^21^ but the energy penalty for grinding is substantial.^29^ In the latter, dissolution is slow (>1000 years),^41^ but far less energy is required for comminution.^29^ Furthermore, large particles are easier to handle, transport, and spread as well as useful for integration in coastal zone management schemes, e.g., beach nourishment.^42^

### 2.3 Goal and Scope, Functional Unit, and System Boundary

The main goal of this LCA study is to identify CEW’s carbon and environmental footprint and its potential for CDR. For this reason, a full LCA was conducted. To minimize uncertainties, mean life cycle inventory (LCI) data that represent average technology were used and the preferred LCI database was Ecoinvent, which follows the pedigree approach to derive empirically based uncertainty factors.^43^ Furthermore, ReCiPe 2016, a harmonized life cycle impact assessment (LCIA) method was used, selecting the hierarchist perspective, which is a scientific consensus model, and average weighting.^44^ Even though an uncertainty analysis is beyond the goal and scope of this LCA, the sensitivity of the results in some of the main parameters was examined and discussed in the “Sensitivity Analysis” section. CEW’s main function, as is the case with all CDR, is to uptake atmospheric CO_2_ and store it safely and permanently. Therefore, as a functional unit (FU), the uptake of 1 t CO_2_ from the atmosphere was chosen, enabling comparison with other coastal^23^ or terrestrial^22^ CDRs, which could be applied in parallel to maximize CO_2_ drawdown. To make the analysis easier, CEW was divided into three main stages, i.e., quarrying, comminution, and coastal spreading and their carbon (life-cycle carbon emissions) and environmental footprint were estimated.

**Figure 1:**
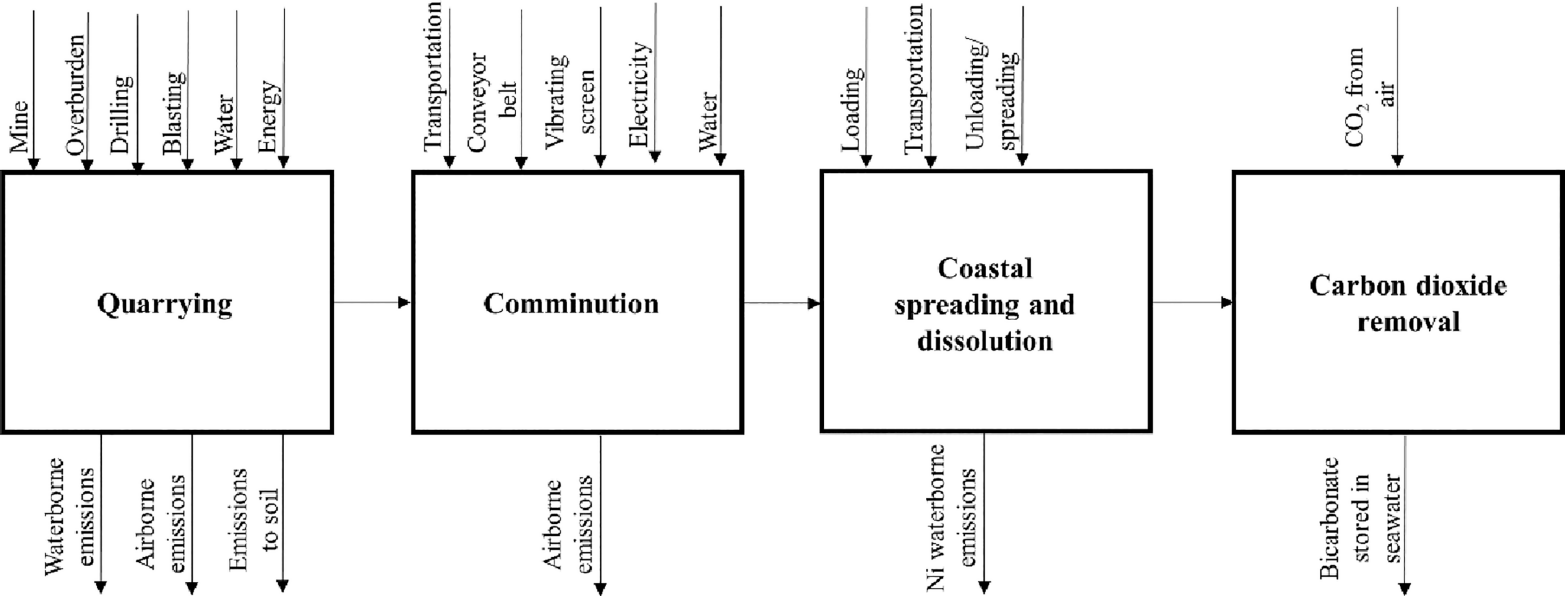
System boundary for coastal enhanced weathering.

All main energy and material flows, along with airborne, waterborne, and soil emissions, were considered (Figure 1). Specifically, it was assumed that olivine is mined from open pits, and the mined area, drilling, blasting, overburden management, and the use of relevant machinery and infrastructure were considered. Furthermore, the relevant airborne, waterborne, and soil emissions from rock mining, e.g., dust from drilling and emissions from the use of explosives, were also considered. The mined rocks are loaded into mine trucks and transported to a rock crushing plant. Only the energy inputs were considered for crushing/grinding, while the machinery, as a material, was external to the system boundary since this is not expected to have a large influence on the results. Water inputs for dust suppression and rock mill cooling were also included in the analysis. However, the generated wastewater was external to the system boundary since cooling water is typically lost through evaporation to the atmosphere, while the water used for dust suppression is directly released to the soil. Finally, both the transportation of pulverized olivine and its spreading in the coastal zone were inside the system boundary. The collected LCI data are based on average technology and therefore have a wide geographical coverage, e.g., the data for the mining activities (drilling, loading, unloading, etc.). The spatial extent was restricted to Europe by assuming that the electricity consumed for olivine comminution originates from Europe’s average electricity mix. In the “Sensitivity Analysis” section, hydropower (renewable energy), which is less restrictive in terms of geographical delimitation, was also considered.

### 2.4 Life Cycle Inventory

In LCI, the processes that were included in the system boundary are quantified and normalized per FU.^26,27^ The collected LCI data for CEW are listed in Table S1. Specifically, it was assumed that an area of 200,000 m^2^ will be mined over 50 years, with an annual capacity of 500 kt,^45^ i.e., 0.5 m^2^ for land use and 0.01 m^2^ per year for land use change were ascribed per FU. Olivine’s Ni content was 0.263%.^28^ An average topsoil thickness of 0.2 m, with a bulk density of 1.3 t m^–3^, was considered,^46^ suggesting that 1.04 kt of overburden is produced year-round. For overburden handling, a bulldozer and a hydraulic shovel were considered, with the energy input taken from US Department of Energy,^47^ corresponding to activities in a surface limestone mine, whereas 200 km was ascribed for overburden transportation, used for backfilling. The quarry infrastructure was assumed to be similar to that of a gravel/sand quarry, as described in Kellenberger et al.^45^

For rock drilling, the diesel and lubricating oil consumption were considered, as described in Rosado et al.^48^ For dust control, a mean energy of 0.45 kWh m^–3^ was ascribed for (borehole) water pumping (diesel-powered).^49^ For blasting, explosives consumption ranges from 0.145 kg (basalt) to 0.374 kg (granite) per extracted t,^46^ with the higher value considered herein since this leads to better fragmentation, which is beneficial for comminution. The rocks are then loaded into mining trucks by means of a hydraulic excavator, with the diesel and lubricating oil consumption taken from Rosado et al.^48^ A 10 km transportation distance was ascribed from the mine to the rock crushing plant by means of a mining truck. The water and energy required for road dust suppression was taken from Gunson.^49^ For the machinery used in quarrying (drill, service trucks, bulk trucks, and graders), Ecoinvent’s data for diesel burned in agricultural machinery, i.e., for running a tractor with a trailer, was used as a proxy.

For the crushing plant, the energy input for the vibrating feeders, screens, and conveyor belts was taken from Lefebvre et al.^22^ The energy input for rock crushing to 1000 μm was taken from Renforth,^50^ while the energy required for grinding to 100, 10, and 1 μm was approximated using data from Moosdorf et al.,^51^ with the latter value being an extrapolation from the published data and therefore associated with higher uncertainty. The water consumption for mills crushing/grinding and dust suppression was taken from Gunson.^49^ All energy inputs for rock comminution were taken as electricity from the average European energy mix. The crushed/pulverized olivine is then loaded in trucks, with the energy required for loading taken from Rosado et al.^48^ and transported, for an average of 50 km, to the coastline where it is spread by a lime spreader.

### 2.5 Life Cycle Impact Assessment

In LCIA, the collected LCI data are translated and linked to specific environmental impacts and damages, which can help identify the environmental sustainability and main environmental hotspots of the system under study. LCIA comprises six different steps: (i) selecting the impact categories that are relevant to the system under study; (ii) allocating the elementary flows that are included in the system boundary to the selected impact categories (classification); (iii) estimating the environmental impact of each category using characterization factors (characterization); (iv) normalizing the identified impact(s) relatively to a spatial and temporal reference(s) (normalization); (v) ranking/sorting the characterized or normalized scores into one or more groups (grouping); and (vi) multiplying the normalized results with weighting factors to assess the importance of each examined impact category relative to the other categories, while the weighted results can also be aggregated into a single score (weighting).^52^ In LCA, the first three steps are mandatory, while the last three are optional.

To convert and quantify the collected LCI data (raw materials, energy, and emissions) into the selected impact categories and then to group, normalize, and/or weight them and express the results into fewer damage categories or into a single score (endpoint level), LCIA methods are used. Here, ReCiPe 2016, a multi-issue LCIA method that can express results both at the midpoint and endpoint levels,^44,53^ was used. At midpoint level, ReCiPe expresses results at 18 impact categories using different characterization factors. For example, the midpoint impact category global warming (GW) (or climate change), reflects the additional radiative forcing over a specific timespan (in kg CO_2_eq), whereas ozone depletion, which can cause damage to human health due to increased UVB radiation, reflects potential releases of trichlorofluoromethane (CFC-11 eq). ReCiPe’s midpoint impact categories are listed in Table 1, while for more details, the reader is referred elsewhere.^44^

**Table 1:** Score of CEW’s Main Stages on Each of ReCiPe’s 2016 Midpoint Impact Categories when Using Silt-Size (10 μm) Olivine

impact category	abbrev.	unit	quarrying	comminution	coastal spreading	total
global warming	GW	kg CO_2_eq	4.5	39.6	6.6	50.7
stratospheric ozone depletion	SOD	g CFC11eq	0.021	0.021	0.004	0.047
ionizing radiation	IR	kBq Co-60 eq	0.093	19.59	0.16	19.843
ozone formation, human health	OF_HH_	kg NO_*X*_ eq	0.18	0.08	0.02	0.29
fine particulate matter formation	FPMF	kg PM_2.5_eq	0.035	0.062	0.010	0.11
ozone formation, terrestrial ecosystems	OF_TE_	kg NO_*x*_eq	0.19	0.084	0.022	0.29
terrestrial acidification	TA	kg SO_2_eq	0.128	0.150	0.017	0.29
freshwater eutrophication	FE	kg Peq	0.001	0.039	0.001	0.04
marine eutrophication	ME	g Neq	3.1	0.12	2.9	0.055
terrestrial ecotoxicity	TE	kg 1,4-DCB	9.5	31.8	138.6	179.9
freshwater ecotoxicity	FET	kg 1,4-DCB	0.18	1.76	0.13	2.1
marine ecotoxicity	MET	kg 1,4-DCB	0.24	2.34	1765.7	1768.3
human toxicity (carcinogenic)	HTc	kg 1,4-DCB	0.45	3.1	19.6	23.2
human toxicity (non-carcinogenic)	HTnc	kg 1,4-DCB	10.4	56.3	10.8	77.5
land use	LU	m^2^a crop eq	1.3	7.1	0.6	9
mineral resource scarcity	MRS	kg Cu eq	0.027	0.064	0.015	0.11
fossil resource scarcity	FRS	kg oil eq	1.083	10.886	2.355	14.323
water consumption	WC	m^3^	0.117	0.736	0.014	0.866

At the endpoint level, normalization and weighting are used and results can be further aggregated into three damage categories, namely, damage to (i) human health, expressed in disability-adjusted life years (DALYs), i.e., the lost years or the years that a person is disabled due to disease or accident; (ii) ecosystem quality, expressed in species year, i.e., local species loss integrated over time; and (iii) resource scarcity (expressed in USD ($)), i.e., the future extra costs incurred for mineral and fossil resource mining.^44^ These three damage categories can be further aggregated into a single score, which in ReCiPe is expressed in Ecopoint (Pt) units, where 1000 Pt correspond to the annual environmental load of an average European inhabitant.^54^

### 2.6 Assumptions and Limitations

Secondary mineral formation, which can affect CEW’s CO_2_ uptake capacity,^55^ as well as CEW’s possible co-benefits, such as ocean acidification mitigation and the improvement of primary production through DSi and Fe releases,^21^ are not sufficiently understood by field or laboratory work and therefore were not included in the analysis. To estimate olivine dissolution rates in coastal environments, the shrinking core model was used together with a conservative estimate for the olivine dissolution rate constant as suggested elsewhere.^41^ To avoid the complexity of using marine vessels and specialized equipment for mineral spreading in seawater, the minerals were assumed to be spread on the dry beach width using standard lime-spreading equipment. Alternatively, olivine could be directly unloaded in piles along the coastline and allow waves and tides to distribute the material in the coastal zone, similar to beach nourishment.^42^ However, this might restrict unloading of olivine to certain seasons (e.g., in most cases, winter conditions provide more wave energy for rapid spreading).

Regarding the olivine impurities, here, a mean concentration of 0.263% was considered for Ni,^28^ while Cr and Fe concentrations were not included in the analysis. It has been estimated that 0.059 to 1.4 kg of olivine can be safely spread per m^2^ of seafloor without exceeding the European Ni environmental quality standard (0.147 μM) and posing risks to benthic biota.^34^ Here, following Flipkens et al.,^34^ this number was estimated at 1.3 kg m^–2^ when assuming a mean depth of 10 m, 24 h residence time, 15 °C water temperature, and 10 μm particle size. The selected water temperature is an approximation of seawater temperature in Europe, where Mediterranean countries such as Spain and Italy exhibit much higher water temperatures than Nordic countries such as Norway. In theory, a larger amount of olivine could be spread on the dry part of the coastal zone since this will be rapidly redistributed within the active part of the coastal zone. However, this conservative assumption was used here to contextualize applications where olivine is spread on the nearshore, e.g., using marine vessels, instead of the dry width of the coastal zone. Finally, the negative effects of Ni release to marine ecosystems were considered by assuming that Ni is solely released in seawater and that there are no negative effects to sediment, while other possible negative impacts such as Cr releases, particulate air pollution, and silt pollution (e.g., turbidity) in coastal waters are not included in the analysis. Most likely, Ni would mainly remain in the solid phase and not in seawater,^55,56^ while, depending on olivine particle size, its total release might take hundreds to thousands of years. Therefore, the assumption that all Ni will be released directly in seawater is assumed to cover the effect of other impurities contained in olivine such as Cr.

## 3 Results and Discussion

### 3.1 ReCiPe at the Midpoint Level

ReCiPe’s midpoint results are shown in Table 1, where it can be seen that CEW’s life-cycle carbon emissions (GW category) or CEW’s carbon penalty amount to around 51 kg of CO_2_eq tCO_2_^–1^, with comminution being the main contributor (78%). Therefore, when Europe’s average electricity mix is powering the process, CEW can remove ∼949 kg of CO_2_ from the atmosphere per 1.25 t of olivine spread over a 10 year timespan. Furthermore, it will take only a few months to recapture CEW’s life-cycle carbon emissions considering the small carbon penalty (∼5%) and the fact that around 26% of the silt-size olivine will be dissolved in year 1. The scores of the remaining midpoint impact categories and their corresponding abbreviation and unit are also shown in Table 1.

**Figure 2:**
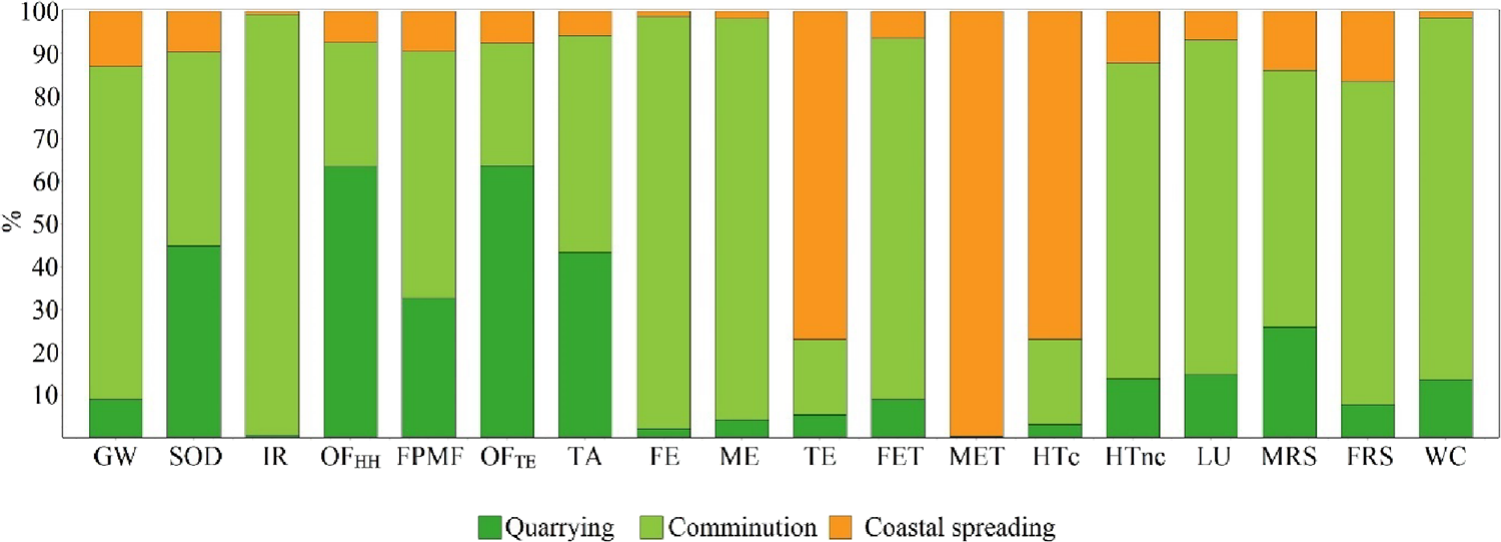
CEW’s contribution on ReCiPe’s 18 midpoint (H) impact categories.

As can be seen in Figure 2, comminution has the largest contribution in most midpoint impact categories. Quarrying is the main contributor in the midpoint impact categories that relate to ozone formation (OF_TE_ and OF_HH_), whereas coastal spreading was the main contributor, by and large, in the MET, HTc, and TE categories (Figure 2 and Table 1). The large contribution of comminution in most midpoint impact categories can be traced back to electricity consumption from Europe’s fossil fuel-dependent average electricity mix and specifically to direct and indirect emissions from fossil fuel extraction, processing, transportation, and combustion for electricity generation. For example, fossil fuel combustion releases harmful and toxic substances to the environment, including heavy metals, polycyclic aromatic hydrocarbons (PAHs), and sulfur oxides (SOx) and nitrogen oxides (NOx), which affect the (eco)toxicity, FPMF (human population intake of PM_2.5_), and TA (absence of species due to soil acidity) impact categories.^54^ Furthermore, the energy industry is water-intensive since water is used across the production chain, spanning from resource extraction to refining to transportation to power generation,^57^ thus affecting WC. In addition, during fossil fuel extraction, processing, and combustion, land is occupied (e.g., coal mines and power stations), while infrastructure and access roads for fossil fuel extraction and transportation also directly affect LU.^58^ The infrastructure for fossil fuel extraction, refining, and combustion makes use of large amounts mineral resources, hence the contribution on MRS, while fossil fuel burning leads to their depletion and, hence, the contribution on FRS. Finally, fossil fuel mining/extraction can expose minerals that contain nitrogen and/or phosphorus, while during combustion SOx, NOx, etc. can be also released,^59^ thus affecting FE and ME. Therefore, the high contributions of the comminution stage across midpoint categories highlights the need for electricity mixes with lower environmental impact, such as renewable electricity.

The large contribution of quarrying to OF_HH_ and OF_TE_ is traced back to blasting and, specifically, to the direct NOx emissions from the nitrogen-containing explosives, i.e., the ammonium nitrate and methylammonium nitrate water-gel explosive (Tovex). Blasting is typically employed in rock excavation due to its high productivity and low cost; however, it also negatively impacts the environment, e.g., through airborne emissions,^60^ and this is also reflected herein. Furthermore, the intensive use of diesel by the machinery employed in quarrying and, particularly, drilling affects most midpoint impact categories for the reasons listed above.

Finally, the large contribution of the coastal spreading stage to the MET and HTc is mainly traced back to Ni emissions to seawater, whereas TET is mainly affected by olivine overland transportation. Specifically, the Ni that is released during olivine dissolution to seawater can pose a risk to marine biota, with toxicological effects occurring when its concentration in an organism exceeds a certain threshold value.^34^ As such, the probable harm in marine environments from increased Ni releases due to olivine spreading and weathering in coastal environments is reflected in the MET score. (Note that the rate of Ni release into seawater was not considered in the LCA and this was treated as a Ni point source.) Furthermore, Ni, as well as other (heavy and transition) metals, are associated with toxicity and carcinogenicity and through bioaccumulation can find their way to humans, and this is reflected on the HTc (e.g., Ni can be carcinogenic) score.^61^ Finally, the contribution to TET is traced back to the use of trucks for overland transportation. Fine particles (PM_10_ and PM_2.5_) are emitted during truck braking, including barium (Ba), copper (Cu), antimony (Sb), and Fe, while tire wear also emits zinc (Zn) particles, which are all known to pose toxic effects on humans and ecosystems.^62^

**Figure 3:**
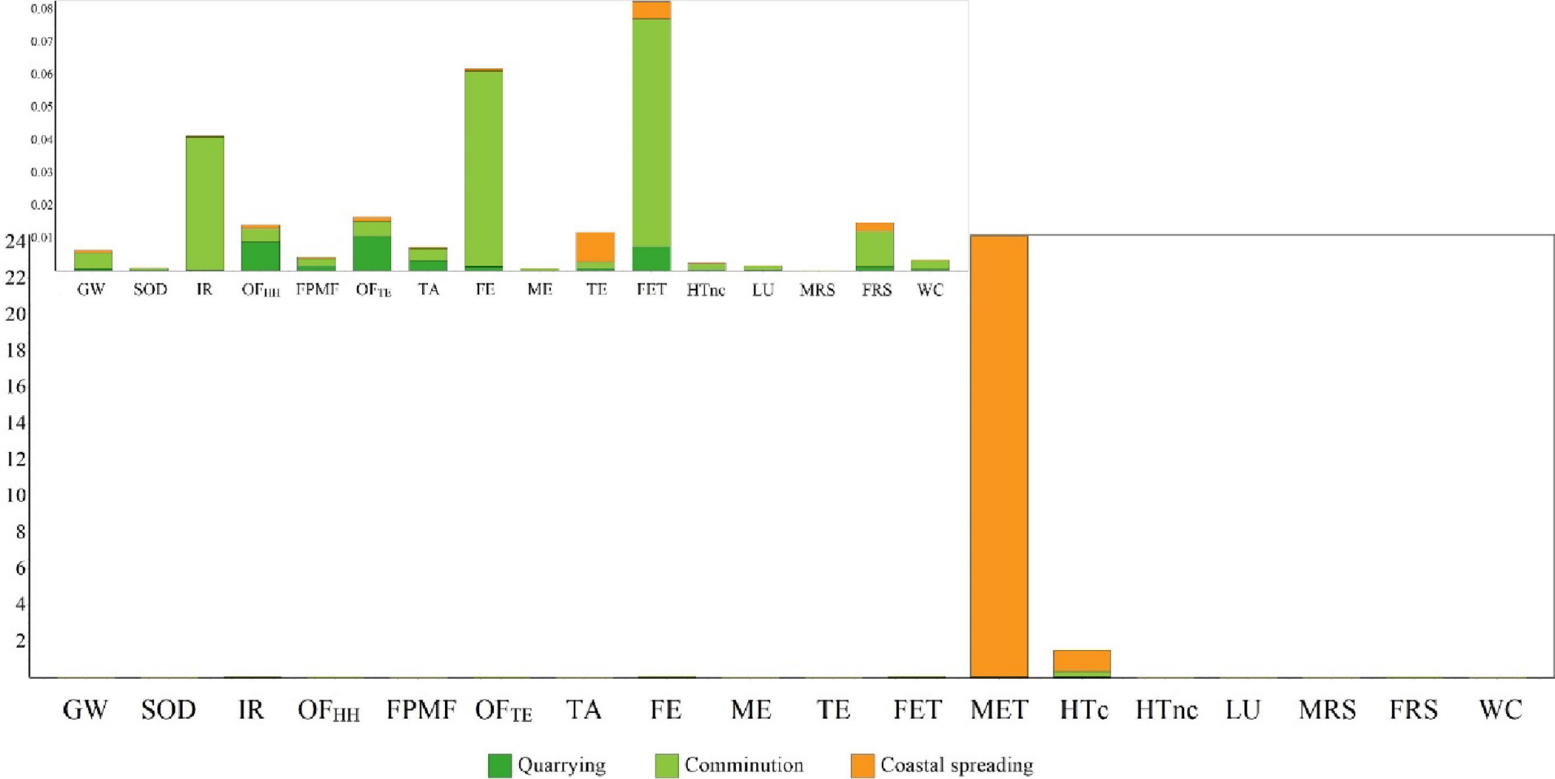
ReCiPe 2016-normalized scores for CEW when using silt-size (10 μm) olivine.

To provide insight about the magnitude of each examined impact category, the results were also normalized using ReCiPe 2016 global normalization factors, i.e., each score was divided with the corresponding score of an average global citizen for the reference year 2010. The normalized results are shown in Figure 3, where it can be seen that the most affected midpoint impact category is the MET, followed to a lesser extent by the HTc. The remaining impact categories yielded low to very low normalized scores (Figure 3, inset). As mentioned above, the MET’s high normalized score mainly traces back to Ni release to marine ecosystems, while that of the HTc is attributed to Ni release and bioaccumulation and, to a lesser extent, to emissions from the fossil fuel-dependent electricity that is mainly consumed during comminution. Therefore, results suggest the importance of olivine composition when considering large-scale CEW projects and the need for low-Ni olivine to avoid the contamination of marine environments.

### 3.2 ReCiPe Results at the Endpoint Level

At the endpoint level, CEW’s total environmental footprint (or environmental penalty) was found to amount to 3.2 Pt tCO_2_^–1^. The comminution stage had the largest contribution (54.1%), followed by coastal spreading (29.5%), whereas quarrying had the lowest contribution (16.4%) (Figure S1). As mentioned above, comminution’s score traces back to electricity consumption from Europe’s average electricity mix and specifically to fossil fuel consumption to produce the electricity, which is responsible for a wide array of environmental impacts, particularly affecting ReCiPe’s midpoint impact categories that relate to the human health damage category. Emissions from olivine mining, primarily from blasting, and from diesel burned in mining activities, are the main contributor to quarrying’s score, again greatly affecting the damage category of human health and, to a much lesser extent, the damage categories of ecosystems and resources. The main contributor to coastal spreading’s score is the release of Ni to seawater (20.9%), followed by olivine transportation (5.6%), while olivine loading and spreading contribute less, mainly due to fossil fuel use as reflected in the human health damage category (Figure S1).

However, when the environmental credits from the removal of 1 t of atmospheric CO_2_ are credited to the system, CEW garners a net negative score of −13 Pt. This suggests that CEW’s environmental impact is outweighed by the benefit of the removed CO_2_. Nevertheless, the environmental penalty is much larger (19.8%) than the carbon penalty (5.1%), and this highlights the need for using multi-issue LCIA methods when assessing CDR approaches rather than only focusing on carbon balances. Furthermore, similar restrictions with other CDR approaches were identified, such as using decarbonized electricity, which is also important for DACS,^24^ while synergies to maximize CO_2_ drawdown could also be available. For example, electricity from BECCS could be used to drive the comminution process, while afforestation/reforestation^25^ of the whole mined area can lead to additional CO_2_ drawdown. It should be also mentioned that CEW’s total carbon and environmental credits will not be gained instantaneously but after months for the silt-size (10 μm) olivine (Figure S2). Finally, the main environmental hotspot was the fossil fuel-dependent electricity that is consumed for olivine comminution (50.6% contribution on CEW’s total environmental footprint), followed by the Ni that is released during olivine weathering (20.9% contribution). This suggest that avenues to further improve CEW’s environmental performance include the use of larger particle sizes and/or more environmentally friendly electricity mixes to drive olivine comminution, as well as low-Ni olivine. Below, these avenues, along with the effect of different transportation distances and means, are examined using scenario analysis.

### 3.3 Sensitivity Analysis

#### 3.3.1 Effect of the Electricity Mix and Transportation Means and Distance

Comminution’s large contribution to CEW’s total environmental footprint is traced back to fossil fuel-dependent electricity. This implies that more environmentally friendly electricity mixes, particularly those with renewable energy, could improve CEW’s environmental profile. For this reason, a scenario was considered where renewable electricity (hydropower) replaces the electricity from Europe’s average energy mix. In this case, the total environmental footprint would be halved, from 3.22 Pt in the base scenario to 1.59 Pt per FU, and comminution’s contribution is significantly lower (7.1% instead of 54.1% in the base scenario). Furthermore, when renewable electricity is used, the importance of restricting Ni releases is highlighted since these amount to 42.4% of the total environmental footprint instead of 20.9% in the base scenario. Finally, in this scenario, blasting, and to a lesser extent overland transportation (truck), also affect CEW’s environmental sustainability of the system since their contributions on the total environmental footprint are 23.5 and 11.7% respectively. To reduce the contribution of blasting, alternative non-explosive rock breakage methods could be employed, such as hydraulic splitting and expansive chemical reagents; however, these are also associated with different challenges and are typically less industrial in nature.^60^

To reduce transportation’s impact, olivine could possibly be mined nearer to the coastal location, i.e., olivine mines next to the coastline. This would reduce the total environmental footprint of the base scenario by only about 5.6% which was transportation’s percentage contribution in the base scenario (50 km). However, if olivine was to be mined further inland, this could significantly impose on CEW’s environmental sustainability. For example, if the transportation distance was increased from 50 to 250 km or to 500 km, then the total environmental footprint would increase by 22.6 or 50.84% respectively (from 3.22 Pt in the base scenario to 3.95 Pt for 250 km and to 4.86 Pt for 500 km transportation distance). Therefore, transportation distance can affect CEW’s environmental sustainability, and this should be considered when olivine is mined from areas that are further inland. In such cases, switching to renewable energy-powered electric trucks or using different means for overland transportation, such as trains, could be considered. For example, if the infrastructure for train transport is available and olivine was transferred by a freight train in Europe, then the total environmental footprint of the base scenario (50 km) would be reduced by 2.08%, while the total environmental footprint of the 500 km transportation scenario would reduce by 13.79%. These reductions are not particularly high since in the base scenario, a high emission standard (EURO 6) but, more importantly, a large (average load factor > 32 t) truck was assumed to be used. If a smaller (average load factor 16–32 t) truck with the same emission standard (EURO 6) was used for olivine overland transportation, then its environmental footprint would increase 78.08%, while if the emission standard was also lower, then it would increase even more (e.g., it would increase by 93.49% if a EURO 5 truck with average load factor 16–32 t was used instead of the EURO 6 32 t truck). The abovementioned data highlights the importance of both transportation distance as well as the means and the specific type of machinery that is used (e.g., emission standard and gross vehicle weight for trucks).

#### 3.3.2 Effect of the Nickel Content and Improved Scenario

In the base scenario, the mean Ni concentration was 0.263%,^28^ which is similar to other estimates (Table S2).^55,63^ However, Ni concentrations can vary from as high as 0.44% (worst-case scenario) to as low as 0.003% (best-case scenario).^35^ In the worst-case scenario, CEW’s total environmental footprint is 3.7 Pt (16% increase compared to the base scenario) and Ni releases contribute 30.7% to the total environmental footprint. In the best case, the total environmental footprint is 2.5 Pt (22% decrease compared to the base scenario) and Ni contribution is practically non-existent. Therefore, as the electricity decarbonizes, the Ni contribution will be much larger, further highlighting the importance of using low-Ni olivine in future CEW schemes. Finally, when hydropower is used along with low-Ni olivine (improved scenario), then CEW’s total environmental footprint reduces by 71%, from 3.2 Pt in the base scenario to 0.92 Pt. In this case, the reduction is traced back to both the lower emissions from hydropower instead of grid electricity as well as from the lower Ni emissions to marine ecosystems. (Note that the Ni releases are not associated with carbon emissions.)

#### 3.3.3 Effect of the Olivine Particle Size

**Figure 4:**
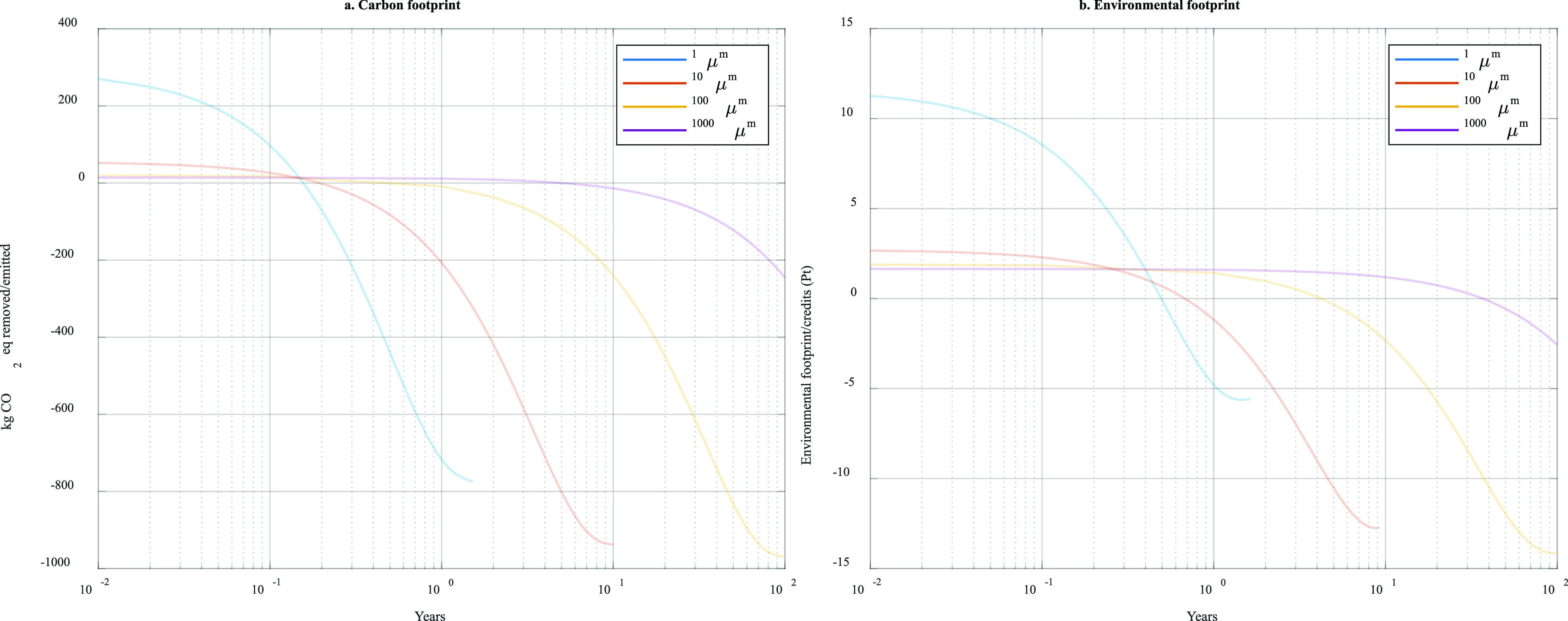
CEW’s life-cycle (a) carbon and (b) environmental footprint and removals for different examined particle sizes over a 100 year timespan (*x* axis in common logarithmic scale).

Due to energy intensity, it is expected that smaller particle sizes will be associated with higher total carbon and environmental footprints. However, the particle size also affects the atmospheric CO_2_ uptake rate since larger particles will require more time to be dissolved. For this reason,focus was also placed on the carbon and environmental footprints of different particle sizes (1, 10 (base scenario), 100, and 1000 μm) and on the time that is required to breakeven on the corresponding footprint, i.e., the moment when CEW becomes carbon and environmental net-negative. The results are shown in Figure S2 and Figure 4 and suggest that the fully pulverized grain size (1 μm) is associated with high carbon (22.3%) and particularly environmental (65.5%) penalties. Furthermore, small particle sizes could be associated with certain engineering challenges pertaining to their transportation and spreading to the coastal zone along with possible impacts to coastal ecosystems and human health due to particulate and silt pollution, the latter causing a multitude of impacts to local ecosystems including turbidity in coastal waters and reduction in light penetration.

Alternatively, CEW using larger particle sizes consumes less energy during comminution and is thus associated with lower carbon and environmental footprints and penalties. However, the slow dissolution rate of the larger particles greatly imposes on the time that is required for CEW’s total carbon and environmental footprint to breakeven, e.g., nearly four decades to break even the total environmental footprint of the 1000 μm particles (Figure 4). However, it is possible that in high energetic coastal environments, olivine’s dissolution rates could be also higher than the shrinking core model estimates due to hydraulic action, abrasion, and attrition especially with non-spherical grains whose rough edges erode quickly during frequent collisions.^55^ As such, the carbon and environmental penalties of larger particle sizes may be compensated shortly after olivine spreading. Not only this, but larger particle sizes also have the advantage of integration with coastal zone management schemes and particularly with beach nourishment, where large volumes of sand are required to counter erosion.^42^ This could also credit CEW with avoided emissions, however, in such cases, the olivine particles should have a similar size to that of the beach sand, implying that the rate of CO_2_ uptake will depend on the local conditions of the nourishment project. Therefore, more research is required on olivine’s dissolution in coastal environments, while the presented dissolution rates can be considered as a conservative approach for CEW’s CO_2_ uptake rate.

Therefore, the 1 and 10 μm olivine will only require a few months to recapture CEW’s life-cycle carbon and environmental emissions before the process becomes net-negative. On the other hand, even though larger particle sizes (100 and 1000 μm) are associated with much lower carbon and environmental penalties, they require more time to breakeven on their carbon footprint and, particularly, on their environmental footprint. As such, it was identified that smaller particles provide more immediate CO_2_ drawdown but at the expense of the total carbon and environmental footprint, whereas larger particles have lower footprints but also exhibit lower CO_2_ drawdown rates. Although the results suggest slow dissolution, larger particles could potentially undergo more momentous collisions in coastal zones, causing more rapid release of alkalinity in the initial years than expected. Furthermore, larger particles may be more easily incorporated into coastal management schemes (e.g., beach nourishment), thus also crediting CEW with avoided emissions as well as credits for CDR. Alternatively, small particle sizes provide fast dissolution and more carbon removal in the initial years, but they also bring unique engineering challenges and possible impacts on local ecosystems (e.g., particulate and silt pollution), rendering their application more promising for remote, less popular (non-touristic), and non-commercial locations. Overall, olivine’s size could be tailored not only for carbon removal but also for beach nourishment and therefore achieve both avoided emissions and carbon removal, whereas in remote high-energy (e.g., strong currents) nearshore areas, pulverized olivine could be used for fast dissolution and CO_2_ drawdown.

## Supporting Information Available

CEW’s LCI data, ReCiPe 2016 endpoint results, carbon and environmental breakeven times for different particle sizes, and Ni concentration in olivine (PDF)
