# Supplementary material for: Life Cycle Assessment of Coastal Enhanced Weathering for Carbon Dioxide Removal from Air
Source: Environ Sci Technol. 2023 Apr 3;57(15):6169–78. doi: 10.1021/acs.est.2c08633 (PMC10116589; doi:10.1021/acs.est.2c08633)
Supplement: Supplementary file 1 — es2c08633_si_001.pdf [file es2c08633_si_001.pdf]

SUPPORTING INFORMATION

# Life cycle assessment of coastal enhanced weathering for carbon dioxide removal from air

**Spyros Foteinis*, James S Campbell, Phil Renforth***

Research Centre for Carbon Solutions, School of Engineering and Physical Sciences, Heriot-Watt University, Edinburgh, EH14 4AS, UK

***Corresponding authors**: s.foteinis@hw.ac.uk, p.renforth@hw.ac.uk

## Table of contents

**SI 1: CEW's LCI** ........ S2

**SI 2: ReCiPe 2016 endpoint results** ........ S4

**SI 3: Carbon and environmental break-even times for different particle sizes** ........ S5

**SI 4: Ni concentration in olivine** ........ S6

# SI 1: CEW's LCI

**Table S1**: The life cycle inventory data for coastal enhanced weathering normalized per functional unit (the uptake of 1 t of atmospheric $CO_2$).

| Process | Input | Quantity | Unit | Database process | Reference |
|---|---|---|---|---|---|
| **Quarrying** | | | | | |
| Olivine | Basalt, in ground | 1.25 | t | Input from nature | Own estimate |
| Overburden | Soil | 0.0026 | t | Input from nature | [39] |
| Overburden handling | Bulldozer and hydraulic shovel (diesel burned in machinery) | 0.628 | mL | Ecoinvent | [40] |
| Overburden transportation | Lorry>32 t (EURO 5) | 1.03 | tkm | Ecoinvent | Own estimate |
| Land use change | Transformation from forest | 0.25 | m² | Input from nature | Own estimate |
| Land use change | Transformation from grassland | 0.25 | m² | Input from nature | Own estimate |
| Land use change | Transformation to mineral extraction site | 0.5 | m² | Input from nature | Own estimate |
| Land use | Occupation, mineral extraction site | 0.01 | m²a | Input from nature | Own estimate |
| Land use change (reforestation) | Transformation to forest | 0.25 | m² | Input from nature | Own estimate |
| Land use change (grassland) | Transformation to grassland | 0.25 | m² | Input from nature | Own estimate |
| Quarry infrastructure | Gravel/sand quarry infrastructure | $5x10^{-8}$ | part | Ecoinvent | Own estimate |
| Drilling | Energy input (diesel burned in machinery) | 2.4 | kWh | Ecoinvent | [41] |
| | Lubricating oil | 1.24 | mL | Ecoinvent | [41] |
| | Dust control (water) | 1.12 | L | Input from nature | [41] |
| | Dust control (energy for pumping) | 0.505 | Wh | Ecoinvent | [42] |
| Blasting | Tovex | 0.181 | kg | Input from nature | [41] |
| Mining operations | Trucks, graders, and lighting (diesel burned in machinery) | 0.446 | kWh | Ecoinvent | [40] |
| Loading | Energy input (diesel burned in machinery) | 1.99 | kWh | Ecoinvent | [22,41] |
| | Lubricating oil | 1.24 | mL | Ecoinvent | [22,41] |
| Water use | Road dust suppression (spraying haul roads) | 0.088 | m³ | Input from nature | [42] |

| | | | | | |
|---|---|---|---|---|---|
| | Water pumping (energy) | 50.1 | Wh | Ecoinvent | [42] |
| **Comminution** | | | | | |
| Transportation to crushing plant | Mining truck | 0.15 | kg | ELCD | Own estimate |
| Crushing/grinding | Vibrating feeder | 0.22 | kWh | Ecoinvent | [22,41] |
| (electricity consumption)* | Conveyor Belt | 0.33 | kWh | Ecoinvent | [22,41] |
| | Vibrating Screen | 0.22 | kWh | Ecoinvent | [22,41] |
| | Crushing to 1000 µm | 3.24 | kWh | Ecoinvent | [43] |
| | Grinding to 100 µm | 16.6 | kWh | Ecoinvent | [44] |
| | Grinding to 10 µm | 92.3 | kWh | Ecoinvent | [44] |
| | Grinding to 1 µm | 513 | kWh | Ecoinvent | [44] |
| Water consumption | Cooling water | 0.395 | $m^3$ | Input from nature | [42] |
| | Dust suppression | 0.012 | $m^3$ | Ecoinvent | [42] |
| **Coastal spreading** | | | | | |
| Loading | Energy input (diesel burned in machinery) | 1.99 | kWh | Ecoinvent | [41] |
| Road transport** | Lorry>32 t (EURO 6) | 62.5 | tkm | Ecoinvent | Own estimate |
| Spreading | Lime spreader | 1.25 | t | Ecoinvent | Own estimate |
| Nickel (Ni) | Emissions to seawater (ocean) | 3.29 | kg | Waterborne emission | [26] |

* In the base scenario medium voltage electricity was used, assumed to originate from Europe (market group containing the geography of all medium voltage electricity supplying markets in Europe, including Albania, Austria, Greece, Poland, Switzerland and another 35 countries). In the sensitivity analysis section, the electricity mix that was considered was hydropower, using mean data for run-of-river-plants, storage plants, and pump storage plants for the European context, as included in ecoinvent latest (3.8) version.

** In the base scenario a EURO 6 lorry with an average load factor > 32 t (gross vehicle weight (GVW) 29.96 t) was considered. In the sensitivity analysis section, a smaller truck (16 – 32 t average load factor) with the same (EURO 6) or lower (EURO 5) emissions standard was considered along with train transportation. For the latter, a freight train was considered for Europe (market group containing the geography of Europe).

# SI 2: ReCiPe 2016 endpoint results

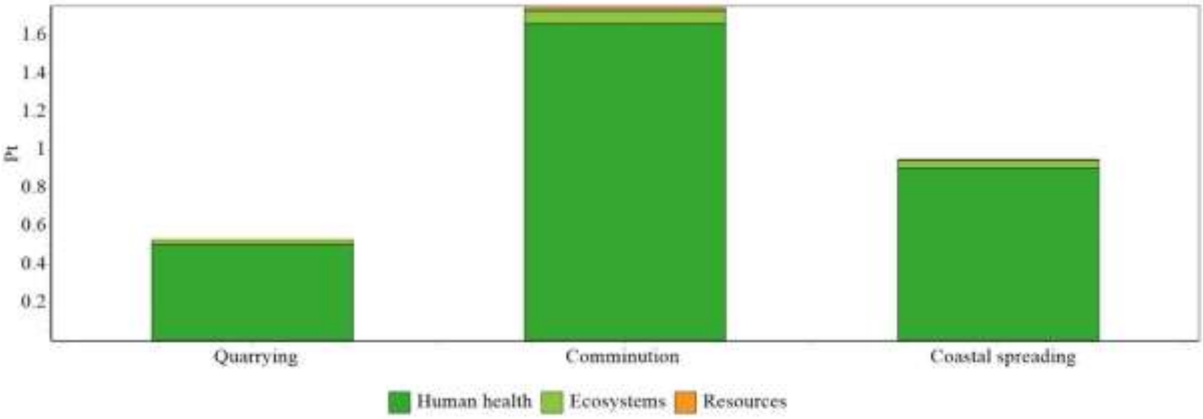


**Figure S1**: ReCiPe 2016 endpoint results for CEW's three main stages.

## SI 3: Carbon and environmental break-even times for different particle sizes

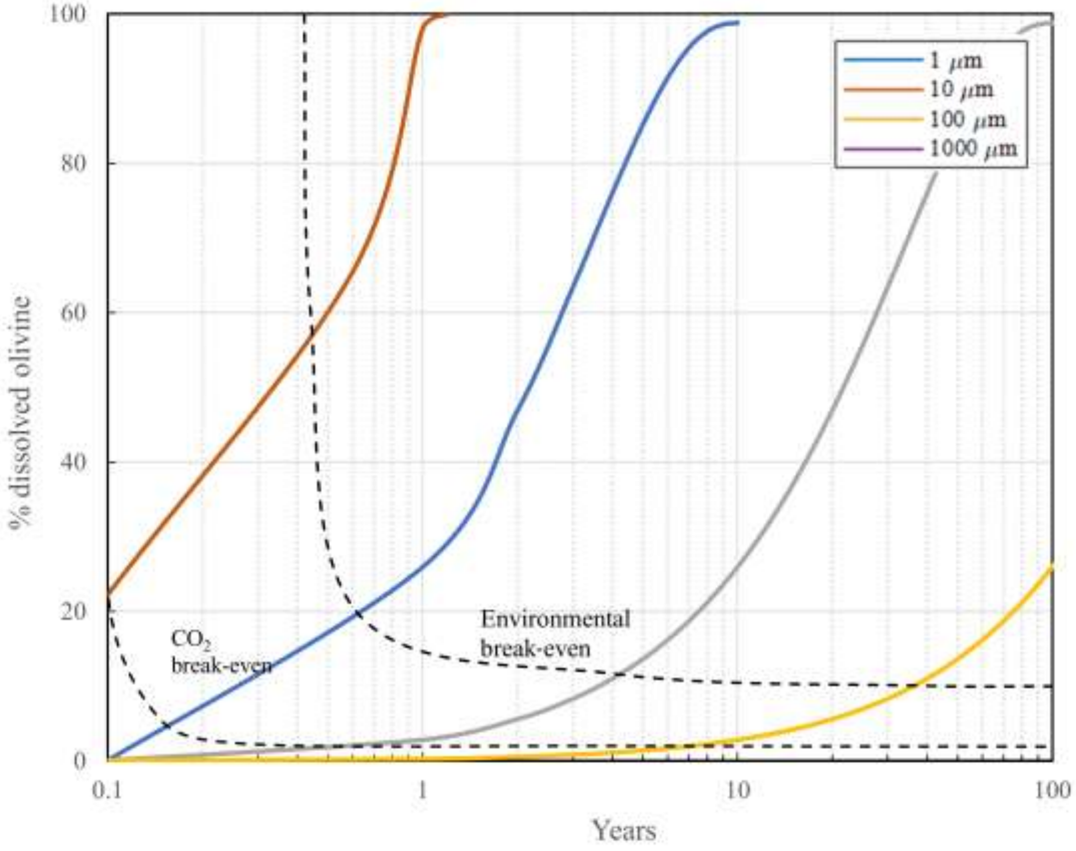


**Figure S2**: Percentage dissolution for different particle sizes of olivine and the corresponding carbon and environmental penalties, i.e., the amount of olivine that needs to be dissolved in time to break-even the carbon and environmental life cycle emissions of coastal enhance weathering before the process becomes net-negative.

## SI 4: Ni concentration in olivine

Table S2 typical Ni concentrations (in wt. %) of various olivine samples taken from the literature. The range is 0.003-0.440 wt. % (Thomson et al. 1984) while the mean Ni concentration for the most recent references (2011 - 2022) is 0.264 wt. %, which is almost identical to the Monserrat et al. (2017) value which was used herein.

| | **Analytical method** | **Element or oxide** | **wt. %** |
|---|---|---|---|
| Chopra and Paterson (1981)[31]* | - | NiO (Ni) | 0.17 (0.133) |
| Thompson et al. (1984)[35]* | WDS | NiO (Ni) | 0.009 – 0.56 (0.003 – 0.440) |
| Jin et al. (1994)[32]* | - | NiO (Ni) | 0.24 (0.188) |
| Keefner et al. (2011)[63] | EPMA | NiO (Ni) | 0.39 (0.306) |
| Santos et al. (2015)[33] | ICP-MS | Ni | 0.27 |
| Montserrat et al. (2017)[28] | ICP-OES | Ni | 0.263 |
| Fuhr et al. (2022)[55] | ICP-OES | NiO (Ni) | 0.33 (0.259) |
| | XRF | NiO (Ni) | 0.28 (0.220) |
| Mean Ni (2011 – 2022) | - | - | 0.264 |

*excluded from determination of the mean.

## SI 5: References


(22) Lefebvre, D.; Goglio, P.; Williams, A.; Manning, D. A. C.; de Azevedo, A. C.; Bergmann, M.; Meersmans, J.; Smith, P. Assessing the Potential of Soil Carbonation and Enhanced Weathering through Life Cycle Assessment: A Case Study for Sao Paulo State, Brazil. *J. Clean. Prod.* **2019**, *233*, 468–481. https://doi.org/10.1016/j.jclepro.2019.06.099.

(26) Montserrat, F.; Renforth, P.; Hartmann, J.; Leermakers, M.; Knops, P.; Meysman, F. J. R. Olivine Dissolution in Seawater: Implications for CO2 Sequestration through Enhanced Weathering in Coastal Environments. *Environ. Sci. Technol.* **2017**, *51* (7), 3960–3972. https://doi.org/10.1021/acs.est.6b05942.

(28) Montserrat, F.; Renforth, P.; Hartmann, J.; Leermakers, M.; Knops, P.; Meysman, F. J. R. Olivine Dissolution in Seawater: Implications for CO2 Sequestration through Enhanced Weathering in Coastal Environments. *Environ. Sci. Technol.* **2017**, *51* (7), 3960–3972. https://doi.org/10.1021/acs.est.6b05942.

(31) Chopra, P. N.; Paterson, M. S. The Experimental Deformation of Dunite. *Tectonophysics* **1981**, *78* (1–4), 453–473. https://doi.org/10.1016/0040-1951(81)90024-X.

(32) Jin, Z. M.; Bai, Q.; Kohlstedt, D. L. High-Temperature Creep of Olivine Crystals from Four Localities. *Phys. Earth Planet. Inter.* **1994**, *82* (1), 55–64. https://doi.org/10.1016/0031-9201(94)90102-3.

(33) Santos, R. M.; Van Audenaerde, A.; Chiang, Y. W.; Iacobescu, R. I.; Knops, P.; Van Gerven, T. Nickel Extraction from Olivine: Effect of Carbonation Pre-Treatment. *Metals* . 2015, pp 1620–1644. https://doi.org/10.3390/met5031620.

(35) Thompson, J. F. H.; Barnes, S. J.; Duke, J. M. The Distribution of Nickel and Iron between Olivine and Magmatic Sulfides in Some Natural Assemblages. *Can. Mineral.* **1984**, *66*, 55–66.

(39) Segura-Salazar, J.; Tavares, L. M. A Life Cycle-Based, Sustainability-Driven Innovation Approach in the Minerals Industry: Application to a Large-Scale Granitic Quarry in Rio de Janeiro. *Miner. Eng.* **2021**, *172*, 107149. https://doi.org/10.1016/j.mineng.2021.107149.

(40) US Department of Energy. Limestone and Crushed Rock. In *TP Mining: Energy and Environmental Profile of the U.S. Mining Industry*; Washington DC, USA, 2013; pp 1–12.

(41) Rosado, L. P.; Vitale, P.; Penteado, C. S. G.; Arena, U. Life Cycle Assessment of Natural and Mixed Recycled Aggregate Production in Brazil. *J. Clean. Prod.* **2017**, *151*, 634–642. https://doi.org/10.1016/j.jclepro.2017.03.068.

(42) Gunson, A. J. Quantifying, Reducing and Improving Mine Water Use. **2013**, 258.

(43) Renforth, P. The Potential of Enhanced Weathering in the UK. *Int. J. Greenh. Gas Control* **2012**, *10*, 229–243. https://doi.org/10.1016/j.ijggc.2012.06.011.

(44) Moosdorf, N.; Renforth, P.; Hartmann, J. Carbon Dioxide Efficiency of Terrestrial

Enhanced Weathering. *Environ. Sci. Technol.* **2014**, *48* (9), 4809–4816. https://doi.org/10.1021/es4052022.

(55) Fuhr, M.; Geilert, S.; Schmidt, M.; Liebetrau, V.; Vogt, C.; Ledwig, B.; Wallmann, K. Kinetics of Olivine Weathering in Seawater: An Experimental Study. *Front. Clim.* **2022**, *4*, 831587. https://doi.org/10.3389/fclim.2022.83158.

(63) Keefner, J. W.; Mackwell, S. J.; Kohlstedt, D. L.; Heidelbach, F. Dependence of Dislocation Creep of Dunite on Oxygen Fugacity: Implications for Viscosity Variations in Earth's Mantle. *J. Geophys. Res.* **2011**, *116* (B5), B05201. https://doi.org/10.1029/2010JB007748.
